# Driving style recognition using machine learning and smartphones

**DOI:** 10.12688/f1000research.73134.1

**Published:** 2022-01-18

**Authors:** Eilham Hakimie bin Jamal Mohd Lokman, Vik Tor Goh, Timothy Tzen Vun Yap, Hu Ng

**Affiliations:** 1 Faculty of Engineering, Multimedia University, Cyberjaya, Selangor, 63100, Malaysia; 2 Faculty of Computing and Informatics, Multimedia University, Cyberjaya, Selangor, 63100, Malaysia

**Keywords:** Machine learning, driver profiling, smartphone, convolutional neural networks

## Abstract

**Background: **The lack of real-time monitoring is one of the reasons for the lack of awareness among drivers of their dangerous driving behavior. This work aims to develop a driver profiling system where a smartphone’s built-in sensors are used alongside machine learning algorithms to classify different driving behaviors.

**Methods:** We attempt to determine the optimal combination of smartphone sensors such as accelerometer, gyroscope, and GPS in order to develop an accurate machine learning algorithm capable of identifying different driving events (e.g. turning, accelerating, or braking).

**Results:** In our preliminary studies, we encountered some difficulties in obtaining consistent driving events, which had the potential to add “noise” to the observations, thus reducing the accuracy of the classification. However, after some pre-processing, which included manual elimination of extraneous and erroneous events, and with the use of the Convolutional Neural Networks (CNN), we have been able to distinguish different driving events with an accuracy of about 95%.

**Conclusions:** Based on the results of preliminary studies, we have determined that proposed approach is effective in classifying different driving events, which in turn will allow us to determine driver’s driving behavior.

## Introduction

Driver behavior strongly influences road safety ^1^ and is currently the main contributor to traffic fatalities. Although many recorded incidents are caused by human errors, researchers suggest that drivers who exhibit a more aggressive driving style are more likely to be engaged in an accident on the road ^2^. In 2018, a total of 548,598 cases of road accidents have been recorded in Malaysia with 6,284 of them resulting in fatalities ^3^. To add to that, the Malaysian Institute of Road Safety Research Institute (MIROS) predicts that the number of fatalities will continue to increase up to 10,716 by the end of 2020 ^4^. Driver profiling attempts to understand and monitor the driver’s behaviour in real-time, leveraging a safer and more responsible driving.

Driving style profiling is the process of collecting driving data (e.g., acceleration, braking, speed, turning rate, location) then applying them to classification models in order to generate a score to determine whether their driving style is safe or unsafe. Driver profiling has gained an increased in demand particularly in the insurance field and rental fleets. For example, AXA FlexiDrive rewards their customers with up to a 20% discount off their insurance premiums if they display good driving behaviour ^5^. In the rental car industry, rental companies turn to RentalMatics, an IoT solution to track rental fleets in real time ^6^. A telematics unit (also called black box) is fitted into a car to gather the relevant data needed to monitor and track the vehicle’s location and behaviour ^7^.

Today’s smartphone is embedded with advanced motion sensors which is suitable for data collection. Smartphones have access to a suite of advanced sensors including accelerometer, gyroscope, magnetometer, gravity sensors and GPS. Previous works like ^8– 11^ proved that if smartphones are properly calibrated, they could be viable alternatives to the conventional telematics unit for monitoring driver behaviour.

This research aims to create a viable system to gather data through a mobile device and apply machine learning algorithms to classify different types of driving events. The research will explore the data gathering phase, where a smartphone will be used as a data collection device to replace the telematics device. Initial calibration of the motion sensors will be applied to ensure the most accurate reading of data for any vehicle. Machine learning algorithms will then be trained and tested to ensure the accuracy in determining different driving events such as careful, normal, careless, and dangerous.

## Methods

This project was divided into two main parts; the first part was the data collection part which is to develop an intuitive smartphone application with the correct combination of sensors to gather raw data on basic vehicular movements and the second part is applying machine learning to train and classify different driving patterns.

### Data collection

**Figure 1.**
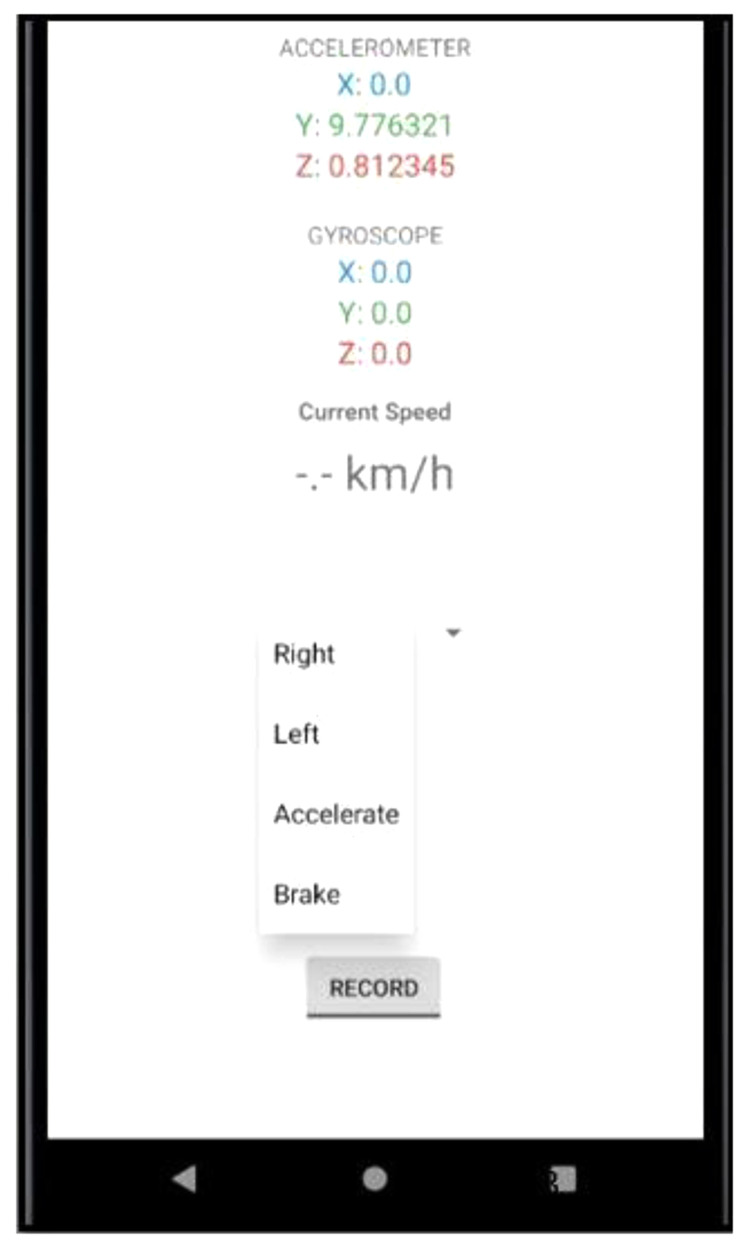
Screenshot of App. The figure shows the user interface of the data gathering app where readings from the smartphone’s accelerometer and gyroscope are updated in real-time.

Data collection utilized the smartphone’s sensors including accelerometer, gyroscope, and GPS. The accelerometer’s readings ( *x*, *y* and *z* axes) relative to the phone position were recorded every 100ms for the duration of the data collection. The gyroscope sensor was used to measure the vehicle’s rate of rotation while the GPS was used to calculate the speed of the vehicle. Figure 1 shows the user interface of the smartphone app. The app recorded several driving event such as:

- Right turns (90°)
- Left turns (90°)
- Acceleration
- Braking

The driving events were attempted at a constant speed of 30km/h. For each driving event, 30 sets of readings were collected in total.

### Data classification

The classification technique employed to assess the implementation of this approach was the 1-Dimensional Convolutional Neural Network (CNN) and the CNN model was built using Tensorflow and Keras. Prior to fitting the machine learning model with data, the data was pre-processed beforehand to overcome problems such as unbalanced data or redundant data which could cause overfitting of model.

Pre-processing began by balancing the data to ensure that the data points for all driving events are equal to prevent skewing of results. Data standardization was also implemented before model fitting. This step is vital to ensure that the model can handle lower valued numbers as the data from the sensors has fluctuations which would cause the result to be inaccurate and require more processing to compensate. Data standardization ‘minimizes’ the amplitude of the data into a form that is much easier to handle by the computer. Frame preparation was done during the pre-processing stage where the frame size was selected to be four seconds and the hop size was chosen to have an overlap of 24 data points. A total of 240 frames were created to accommodate the sample size. The sample data was then split into 80% for training set and 20% testing set.

**Figure 2.**
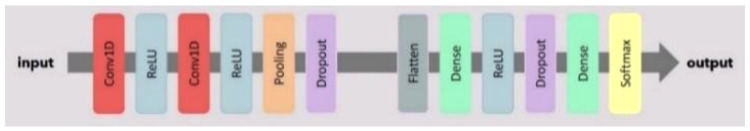
1-D Convolution Neural Network. The illustration above shows the various layers used to develop the machine learning model. The various layers include Conv1D layers, Rectified Linear Unit (ReLU) Activation function, Pooling layers, Dropout, and Dense layers.

After pre-processing, the CNN layer was built. The neural network layer was built following multiple trial and errors to ensure the outcome produces a good-fit model graph. An initial 1-dimensional CNN layer called ‘Conv1D’ was created. The layer develops a convolutional kernel that convolves with the layer input over a single dimensional space to produce a tensor of outputs. Following the initial layer, an activation layer called the Rectified Linear Unit (ReLU) was applied as it is the ideal activation layer for Multilayer Perceptron (MLP) and CNN. ReLU is an activation function that utilizes simple arithmetic that will output the value directly if it is more than 0.0, and will output 0.0 if the input is less than 0.0 ^12^. After that, a pooling layer by the name of ‘MaxPool1D’ was applied to reduce the number of feature maps by taking the maximum value over a certain pool size. This layer is recommended in CNN models as it reduces variance and minimizes computations ^13^. A ‘Dropout’ layer is then applied which operates by setting the outgoing edges of each node to zero to minimize overfitting ^14^. A ‘Dense’ layer is applied to allow the neurons in the ‘Dense’ layer to receive input from neurons of the previous layer ^15^. Finally, a ‘Softmax’ layer is applied to convert the output into a probability distribution. Figure 2 visualizes the 1-dimension CNN used for this research.

## Results and discussion

**Figure 3.**
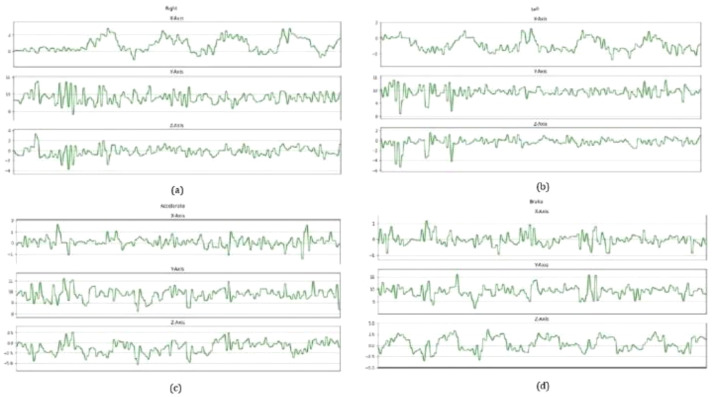
Accelerometer readings. ( **a**) The *x*-axis of the accelerometer form peaks at each Right turning. ( **b**) Left turn creates an opposite pattern from Right turns as each turning point forms successive valleys upon the *x*-axis. ( **c**) Accelerate event creates valleys on the *z*-axis of the accelerometer. ( **d**) Brake event indicates an opposite pattern where peaks are formed on the *z*-axis of the accelerometer at each braking point.

The readings from the smartphone’s accelerometer for the various driving events are shown in Figure 3. Due to the vibrations of the vehicle during idling and during movements, a few spikes in the amplitude can be seen. The spikes in the accelerometer readings during idling could also be caused by the vehicle engine running.

The different types of driving events that were demonstrated could be easily distinguished between each other. In Figure 3(a), the accelerometer readings for the *x*-axis displays multiple peaks successively. Each peak in the graph translates to a right turn where the highest point of each peak translates to the highest force during each turn.

On the other hand, the readings for left turn events show the opposite result where in Figure 3(b), the accelerometer readings for the *x*-axis displays multiple valleys successively. Each valley translates to a single left turn in real life. However, Figure 3(a) and 3(b) display a close resemblance in the *y* and *z*-axis during the early phase of the recording, where the *y* and *z*-axis shows multiple peaks followed by multiple valleys. This could possibly be caused by the vibrations coming out from the engine of the car moving from a stationary position.

In Figure 3(c), the accelerometer values show readings for the vehicle during acceleration. A series of valleys are formed in the *z*-axis where each valley translates to an acceleration event being performed. The intensity of the valley is not very pronounced as the throttle was gradually applied to ensure smooth acceleration. If the accelerator was pressed more aggressively, we predict the *z*-axis value of the accelerometer to be more pronounced. Figure 3(d) shows the accelerometer readings for the vehicle under braking events. The output is the opposite of acceleration event, as the *z*-axis of the accelerometer form peaks at each point of braking. Each peak is much more visually defined as the brakes were applied much more aggressively to fully stop the car from 30km/h. In both Figure 3(c) and 3(d), the *x* and *y*-axis produced a few peaks followed by valleys. This could be caused by the inertia acting on the vehicle or the engine vibrations in each driving event.

As observed from the accelerometer plots, each driving event could be distinguished from each other as each event has their specific features. For left and right turns there is a distinct difference in their *x*-axes while acceleration and braking events can be distinguished in the *z*-axis. The difference in features for each driving event could be highly advantageous as we could train machine learning algorithms to identify their specific features and automatically categorize each of the driving events.

**Figure 4.**
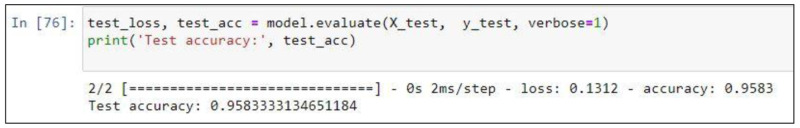
Model Evaluation. The figure visualizes the evaluation results for the model when classifying the testing set where an accuracy of 95.83% was obtained.

The machine learning experiment was carried in two phases, training phase and test phase. 80% of the total sample data was used to train the 1-CNN model and 20% of the sample data was used to test the accuracy of the trained model. After numerous experiments, it is discovered that the most optimal epoch value is 100 combined with a batch size of 32 which produced a model accuracy of 95.83%. Figure 4 shows the test results of the machine learning model ^16^.

**Figure 5.**
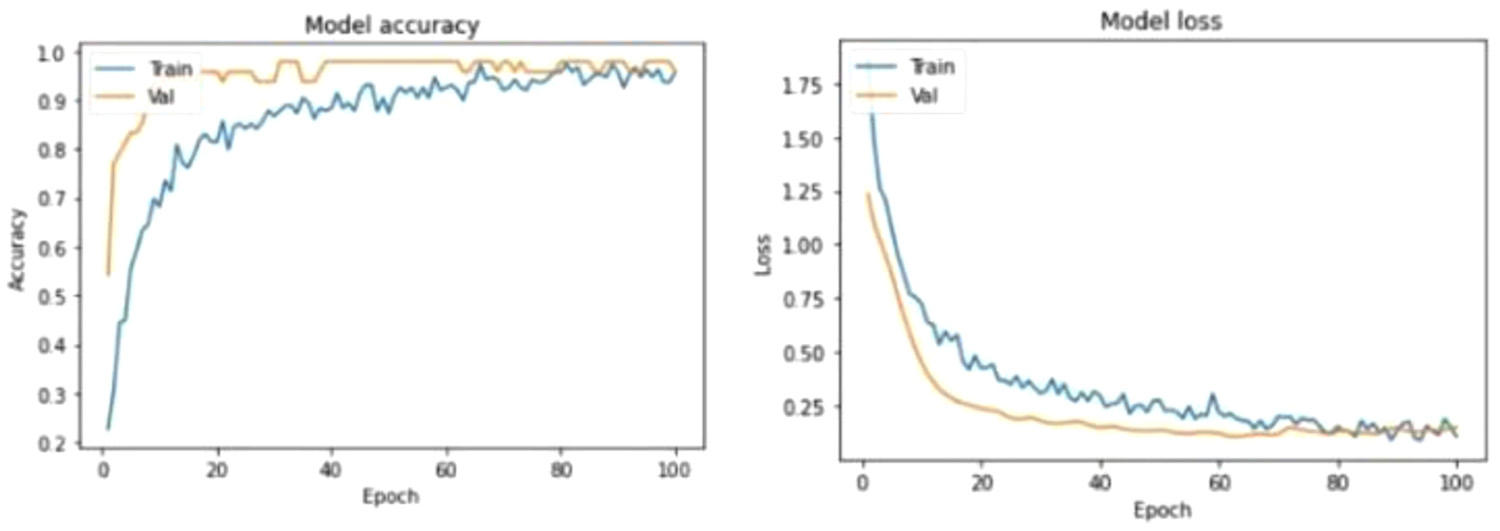
Learning Curves. The figure depicts the learning curves for Model Accuracy and Model Loss where each graph has a curve for Training and Validation performances.

The Model Loss Curve in Figure 5 shows that the training loss decreases as the epoch value increases. Upon reaching epoch value 80, the training loss stops decreasing and stabilizes. Similarly, the validation loss decreases as the epoch value increases but stabilizes at around 80 epochs. It can be observed that the validation loss and training loss has very minimal gap between them which states that the model is a good fit model. Continued training of this model will likely cause the model to be overfitted.

**Figure 6.**
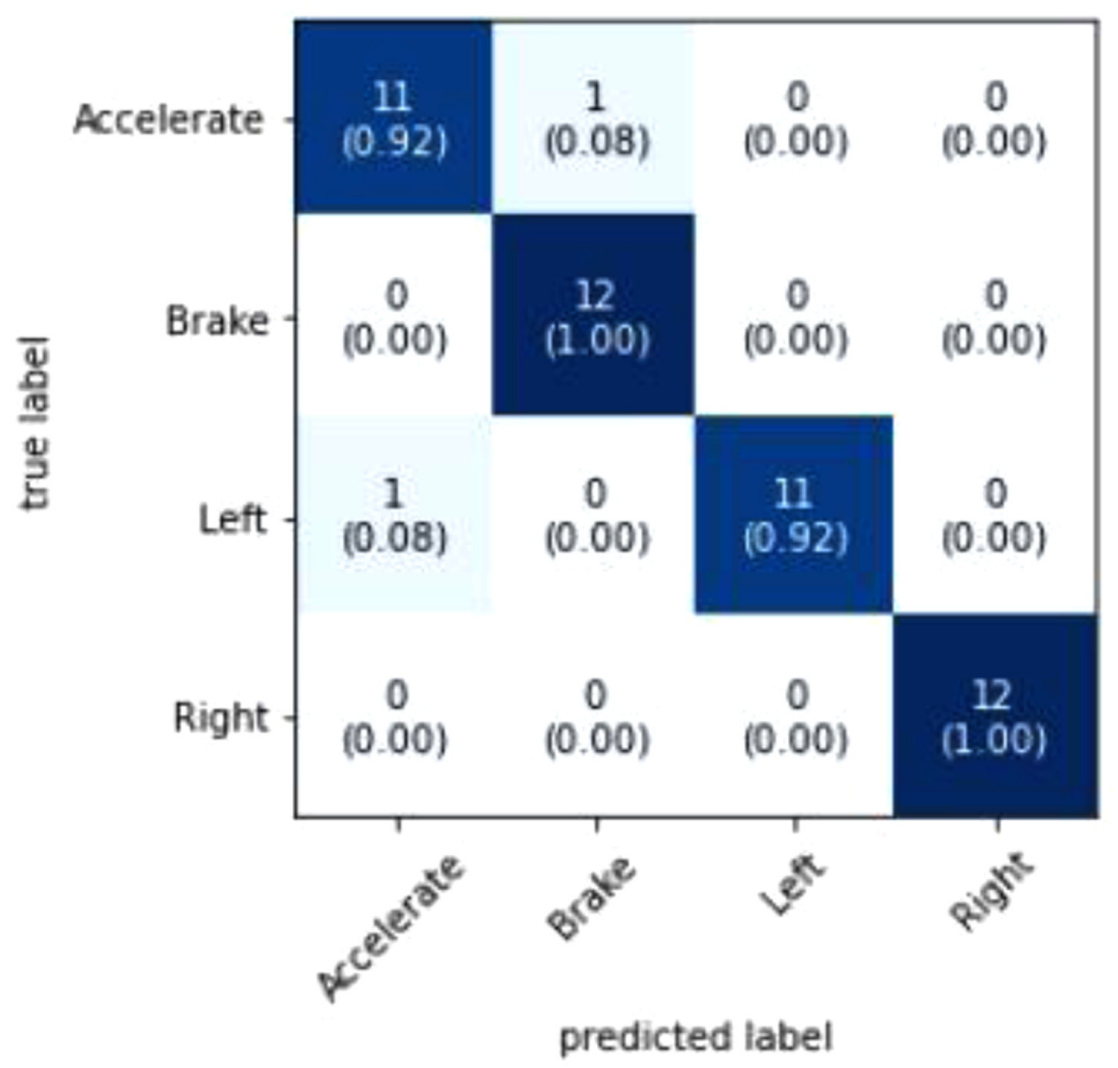
Confusion Matrix. The figure shows the Confusion Matrix where the prediction results are summarized to show the performance of the model.

Figure 6 visualizes that out of the model predicted 46 out of 48 events correctly. The two events that were wrongly predicted was an Accelerate event which was supposed to be a Left turn event and a Braking event which was supposed to be an Accelerate event. A probable reason as to why the model predicted these events wrongly is because of high variance of data because of the difficulty in maintaining consistency in simulating the driving events. Additional pre-processing of data could also improve the accuracy of the model as it removes redundant data and noise. Increasing the epoch value could also further improve the accuracy but caution must be kept in mind to make sure that the model is not overfitted.

Additional work could be done to improve the quality of data samples. For example, investing on a phone mount that does not wobble too much under engine vibrations. The data gathering framework could also be improved to ensure that only the relevant data samples are collected and not redundant data. For the application side, the sampling rate could also be tweaked to ensure that redundant accelerometer and gyroscope readings are not recorded.

## Conclusion

Smartphone sensors have improved massively over the years and prove to be a viable option when used as a data gathering apparatus to monitor driver behavior. In turn, the collected data could then be processed using machine learning algorithms to classify different driving events with high reliability. For this research we have applied the concept of Convolutional Neural Networks which works effectively in classifying different driving events with an accuracy of 95.83%.

For our future work, we will continue to collect data for different driving events such as aggressive driving and careless driving. Additionally, we will feed the collected data into the machine learning model to allow it to classify an even more diverse palette of driving events. Next, we will consider adding a calibration feature into our data gathering application to allow accurate data collection without mounting the phone to a phone mount which will further improve user experience. As there are many machine learning models suitable for classifying time-series sensor data, we will experiment with different types of machine learning models to find out which will provide the best accuracy. Finally, we will deploy the machine learning model onto a smartphone to allow real-time classification of driving style. With these steps planned, it could potentially enhance the impact of this research in terms of traffic safety which was the main goal of this paper.

## Data Availability

Harvard Dataverse: Driving Events, https://doi.org/10.7910/DVN/F5JZHF ^16^.

This project contains the following underlying data:

- driving_events.db (Raw data from sensors)

Data are available under the terms of the Creative Commons Zero "No rights reserved" data waiver (CC0 1.0 Public domain dedication).
