# Supplementary material for: Driving event recognition using machine learning and smartphones
Source: F1000Res. 2022 Dec 19;11:57. Originally published 2022 Jan 18. [Version 2] doi: 10.12688/f1000research.73134.2 (PMC10111143; doi:10.12688/f1000research.73134.2)
Supplement: Peer review history

# Peer review: Reviewer response for version 2

**DOI:** 10.5256/f1000research.142216.r158423

**Published:** 2023-04-17

**Authors:** Muhammad Reza Z'aba

**Affiliations:** 1 Mimos Berhad, Kuala Lumpur, Federal Territory of Kuala Lumpur, Malaysia

Authors have sufficiently made amendments to previous comments.

Is the work clearly and accurately presented and does it cite the current literature?

Partly

If applicable, is the statistical analysis and its interpretation appropriate?

Not applicable

Are all the source data underlying the results available to ensure full reproducibility?

Partly

Is the study design appropriate and is the work technically sound?

Partly

Are the conclusions drawn adequately supported by the results?

Partly

Are sufficient details of methods and analysis provided to allow replication by others?

Partly

Reviewer Expertise:

Information security, cryptography

I confirm that I have read this submission and believe that I have an appropriate level of expertise to confirm that it is of an acceptable scientific standard.

# Peer review: Reviewer response for version 2

**DOI:** 10.5256/f1000research.142216.r158424

**Published:** 2023-01-11

**Authors:** Babul Salam KSM Kader Ibrahim

**Affiliations:** 1 Faculty of Engineering, Environment and Computing, Coventry University, Coventry, UK

I am satisfied with the author's responses to my questions/issues raised in my initial review. Thanks

Is the work clearly and accurately presented and does it cite the current literature?

Yes

If applicable, is the statistical analysis and its interpretation appropriate?

Yes

Are all the source data underlying the results available to ensure full reproducibility?

Yes

Is the study design appropriate and is the work technically sound?

Yes

Are the conclusions drawn adequately supported by the results?

Yes

Are sufficient details of methods and analysis provided to allow replication by others?

Partly

Reviewer Expertise:

Sensors, Modelling and Control

I confirm that I have read this submission and believe that I have an appropriate level of expertise to confirm that it is of an acceptable scientific standard.

# Peer review: Reviewer response for version 1

**DOI:** 10.5256/f1000research.76760.r143359

**Published:** 2022-07-08

**Authors:** Babul Salam KSM Kader Ibrahim

**Affiliations:** 1 Faculty of Engineering, Environment and Computing, Coventry University, Coventry, UK

This paper discusses the attempt to use the smartphone sensors such as accelerometer, gyroscope, and GPS in order to develop an accurate machine learning algorithm capable of identifying different driving events (e.g. turning, accelerating, or braking). The convolutional neural network (CNN) has been applied to classify these different events.

  

- The title should be corrected as the work recognises the driving events, not the driving style.
- The methodology should be improved by explaining and showing where the smartphone is mounted on the screen or on the dashboard. The illustration may help the reader.
- Mounting on the dashboard is not a good option as there are vibrations due to the car’s engine. This should be avoided.
- The labels in Figure 3 are not very clear.

  

 Please proofread the document before submission. There are too many grammatical mistakes and need to be addressed as follows-

 Replace

 Conclusions: Based on the results of preliminary studies, we have determined that the proposed approach is effective in classifying different driving events, which in turn will allow us to determine driver’s driving behavior.

 with

 Conclusions: Based on the results of preliminary studies, we have determined that **the** proposed approach is effective in classifying different driving events, which in turn will allow us to determine driver’s driving behavior.

 Replace

 Driver profiling attempts to understand and monitor the driver’s behaviour in real-time, leveraging a safer and more responsible driving.

 with

 Driver profiling attempts to understand and monitor the driver’s behaviour in real-time, leveraging safer and more responsible driving. 

 Replace

 The app recorded several driving event such as: 

 with

 The app recorded several driving **events** such as:

 Replace

 Prior to fitting the machine learning model with data, the data was pre-processed beforehand to overcome problems such as unbalanced data or redundant data which could cause overfitting of model.

 with

 Prior to fitting the machine learning model with data, the data was pre-processed beforehand to overcome problems such as unbalanced data or redundant data which could cause overfitting of **the** model.

 Replace

 The neural network layer was built following multiple trial and errors to ensure the outcome produces a good-fit model graph.

 with

 The neural network layer was built following multiple **trials **and errors to ensure the outcome produces a good-fit model graph.

 Replace

 Figure 3. Accelerometer readings. (a) The x-axis of the accelerometer form peaks at each Right turning. (b) Left turn creates an opposite pattern from Right turns as each turning point forms successive valleys upon the x-axis. (c) Accelerate event creates valleys on the z-axis of the accelerometer. (d) Brake event indicates an opposite pattern where peaks are formed on the z-axis of the accelerometer at each braking point.

 with

 Figure 3. Accelerometer readings. (a) The x-axis of the accelerometer form peaks at each Right **turn**. (b) **The** Left turn creates an opposite pattern from Right turns as each turning point forms successive valleys upon the x-axis. (c) Accelerate event creates valleys on the z-axis of the accelerometer. (d) **The** brake event indicates an opposite pattern where peaks are formed on the z-axis of the accelerometer at each braking point.

 Replace

 Figure 3(a) and 3(b) display a close resemblance in the y and z-axis during the early phase of the recording, where the y and z-axis shows multiple peaks followed by multiple valleys.

 with

**Figures **3(a) and 3(b) display a close resemblance in the y and z-axis during the early phase of the recording, where the y and z-axis **show **multiple peaks followed by multiple valleys.

 Replace

 As observed from the accelerometer plots, each driving event could be distinguished from each other as each event has their specific features.

 with

 As observed from the accelerometer plots, each driving event could be distinguished from **the other** as each event has **its** their specific features.

 Replace

 The machine learning experiment was carried in two phases, the training phase, and test phase

 with

 The machine learning experiment was carried **out **in two phases, **the** training phase and **the** test phase

 Replace

 A probable reason as to why the model predicted these events wrongly is because of high variance of data because of the difficulty in maintaining consistency in simulating the driving events.

 with

 A probable reason why the model predicted these events wrongly is because of **the** high variance of data because of the difficulty in maintaining consistency in simulating the driving events.

 Replace

 It can be observed that the validation loss and training loss has very minimal gap between them which states that the model is a good fit model

 with

 It can be observed that the validation loss and training loss have **a** very minimal gap between them which states that the model is a good fit model

 Replace

 Figure 6 visualizes that out of the model predicted 46 out of 48 events correctly. 

 with

 Figure 6 visualizes that the model predicted 46 out of 48 events correctly. 

 Replace

 Next, we will consider adding a calibration feature into our data gathering application to allow accurate data collection without mounting the phone to a phone mount which will further improve user experience. 

 with

 Next, we will consider adding a calibration feature to our data gathering application to allow accurate data collection without mounting the phone to a phone mount which will further improve **the** user experience.

Is the work clearly and accurately presented and does it cite the current literature?

Yes

If applicable, is the statistical analysis and its interpretation appropriate?

Yes

Are all the source data underlying the results available to ensure full reproducibility?

Yes

Is the study design appropriate and is the work technically sound?

Yes

Are the conclusions drawn adequately supported by the results?

Yes

Are sufficient details of methods and analysis provided to allow replication by others?

Partly

Reviewer Expertise:

Sensors, Modelling and Control

I confirm that I have read this submission and believe that I have an appropriate level of expertise to confirm that it is of an acceptable scientific standard, however I have significant reservations, as outlined above.

# Response

**Published:** 2022-12-14

**Authors:** Vik Tor Goh

**Affiliations:** Multimedia University, Malaysia

The title will be revised to “Driving Event recognition using machine learning and smartphones” to better reflect the content of the paper. However, this is subject to the approval of F1000’s editors.

 The smartphone was placed on the center dashboard via a phone mount. The paper has been revised to include this information for better clarity.

 We have already tested different position such as left side of the dashboard, right side of the dashboard, and even on the center console below the radio but came to a conclusion that the center of the dashboard was the best option for this car.

 The labels in Figure 3 have been enhanced for better clarity.

 All suggested amendments have been done. The rest of the document has also been proofread to the best of our ability to minimise grammatical and typological errors. Thank you very much for highlighting the errors.

# Peer review: Reviewer response for version 1

**DOI:** 10.5256/f1000research.76760.r122549

**Published:** 2022-02-28

**Authors:** Muhammad Reza Z'aba

**Affiliations:** 1 Mimos Berhad, Kuala Lumpur, Federal Territory of Kuala Lumpur, Malaysia

The article describes an attempt to use mobile phone as an apparatus to collect data regarding certain driving events such as turning, accelerating or braking. Then, convolutional neural network (CNN) is applied to classify these different events. The authors claim that CNN managed to classify the events with a high accuracy (95.83%).

 I think the title is a bit misleading. The title mentions about **driving style recognition**. However, what the work recognises was the driving **events** such as turning, accelerating or braking. When "style" was mentioned, I was thinking something along the lines of "careless driving" or "careful driving". In essence, I believe the work has yet to recognise the driving **style**, only the driving **events**. It is good if the authors can categorise several driving **styles** according to a set of driving **events**.

 The authors mention that in order to improve data collection, it is proposed to use a phone mount in order to eliminate inaccurate readings due to engine vibrations. I think the authors should do this and include the results in the article since I think a phone mount is not that expensive and is widely available. The results can then perhaps be compared with the data collected without using the phone mount. This is to see how much accuracy can be gained.

 The authors also did not state the model of the smartphone used in the work. It is also good if different smartphone models are used in order to make a comparison.

Is the work clearly and accurately presented and does it cite the current literature?

Partly

If applicable, is the statistical analysis and its interpretation appropriate?

Not applicable

Are all the source data underlying the results available to ensure full reproducibility?

Partly

Is the study design appropriate and is the work technically sound?

Partly

Are the conclusions drawn adequately supported by the results?

Partly

Are sufficient details of methods and analysis provided to allow replication by others?

Partly

Reviewer Expertise:

Information security, cryptography

I confirm that I have read this submission and believe that I have an appropriate level of expertise to confirm that it is of an acceptable scientific standard, however I have significant reservations, as outlined above.

# Response

**Published:** 2022-12-14

**Authors:** Vik Tor Goh

**Affiliations:** Multimedia University, Malaysia

The title will be revised to “Driving **event** recognition using machine learning and smartphones” to better reflect the content of the paper. However, this is subject to the approval of F1000’s editors.

 A phone mount was indeed used to collect data in the current experimental setup. The mount was semi-rigid as this provided a good transfer of energy/vibration from the vehicle as well as some dampening against minor vibrations. It functioned as intended but also introduced some unwanted wobble due to its semi-rigid build. As such, in our future experiments, we will investigate using other models of mounts with different build quality.

 The experiments used a Samsung Galaxy S10 as the data collector. The paper has been revised to include some additional information about the data collection setup. A comparison of different smartphone models was not in the scope of the current phase but will be considered in our future experiments.
